# Proteome-level display by 2-dimensional chromatography of extracellular matrix-dependent modulation of the phenotype of bladder cancer cells

**DOI:** 10.1186/1477-5956-4-13

**Published:** 2006-06-02

**Authors:** Robert E Hurst, Kimberly D Kyker, Mikhail G Dozmorov, Nobuaki Takemori, Anil Singh, Hiroyuki Matsumoto, Ricardo Saban, Edna Betgovargez, Michael H Simonian

**Affiliations:** 1 Department of Urology, Oklahoma University Health Sciences Centre, Oklahoma City, OK 73104, USA; 2 Department of Biochemistry and Molecular Biology, Oklahoma University Health Sciences Centre, Oklahoma City, OK 73104, USA; 3 Department of Occupational and Environmental Health, Oklahoma University Health Sciences Centre, Oklahoma City, OK 73104, USA; 4 Department of Physiology, Oklahoma University Health Sciences Centre, Oklahoma City, OK 73104, USA; 5 Biomedical Research Division, Beckman Coulter Inc. 4300 N. Harbor Blvd., Fullerton, CA 92834, USA

## Abstract

**Background:**

The extracellular matrix can have a profound effect upon the phenotype of cancer cells. Previous work has shown that growth of bladder cancer cells on a matrix derived from normal basement membrane suppresses many malignant features that are displayed when the cells are grown on a matrix that has been modified by malignant tumors. This work was undertaken to investigate proteome-level changes as determined by a new commercially available proteome display involving 2-dimensional chromatography for bladder cancer cells grown on different extracellular matrix preparations that modulate the expression of the malignant phenotype.

**Results:**

Depending on the matrix, between 1300 and 2000 distinct peaks were detected by two-dimensional chromatographic fractionation of 2.1 – 4.4 mg of total cellular protein. The fractions eluting from the reversed-phase fractionation were suitable for mass spectrometric identification following only lyophilization and trypsin digestion and achieved approximately 10-fold higher sensitivity than was obtained with gel-based separations. Abundant proteins that were unique to cells grown on one of the matrices were identified by mass spectrometry. Following concentration, peaks of 0.03 AU provided unambiguous identification of protein components when 10% of the sample was analyzed, whereas peaks of 0.05 AU was approximately the lower limit of detection when the entire sample was separated on a gel and in-gel digestion was used. Although some fractions were homogeneous, others were not, and up to 3 proteins per fraction were identified. Strong evidence for post-translational modification of the unique proteins was noted. All 13 of the unique proteins from cells grown on Matrigel were related to MYC pathway.

**Conclusion:**

The system provides a viable alternative to 2-dimensional gel electrophoresis for proteomic display of biological systems. The findings suggest the importance of MYC to the malignant phenotype of bladder cancer cells.

## Background

The extracellular matrix (ECM) exerts major modulatory effects on the phenotype of both malignant and normal epithelial cells [1-5]. Malignant cells remodel the local ECM, which then becomes permissive for malignant growth [6,7]. In earlier reports from our laboratory we have developed a model for investigating the effect of ECM on cancer cells by growing them on ECM derived from normal tissue, from malignant tissue, and on plastic in conventional tissue culture where ECM effects are essentially absent [8,9]. Matrigel is a gel-forming basement membrane-derived ECM on which bladder cancer cells recapitulate their *in vivo *phenotype. In contrast, on SISgel, a gel-forming ECM product derived from normal porcine small intestine submucosa (SIS), the same bladder cancer cell lines display a much more normalized phenotype in which invasion is suppressed and the lowest grade cell line forms a multi-layered structure reminiscent of normal bladder epithelium. The mechanism for this effect is not known and may reflect protein-level changes not reflected in the transcriptome.

While 2-dimensional gel electrophoresis is a well-established method for investigating the proteome, a significant fraction of the proteome is not reflected in electrophoretograms [10]. The sensitivity is limited, large proteins do not even enter the gel, and the proteins must be purified away from the gel components for mass spectrometry. Two-dimensional chromatography is a relatively novel proteomic approach in which separation by pI and hydrophobicity achieves a proteome-level display [11,12]. We describe the application of 2-dimensional chromatographic proteomics to describing the effect of ECM on the phenotype of bladder cancer cells, including the mass spectrometric identification of some of the major differences in the proteomes of cells grown on Matrigel, SISgel and plastic.

## Methods

### Cell culture

J82 Transitional cell carcinoma cells were obtained from the American Type Culture Collection. (Bethesda, MD) and were grown as previously described [8,9] on plastic, SISgel (Cook Biotech, W. Lafayette, IN) and Matrigel (BD Biosciences, Bedford, MA). Briefly, 0.8 ml of ice cold Matrigel was layered onto polyethylene terephthalate membranes of 6-well cell culture inserts (Falcon, Becton-Dickinson Labware, Franklin Lakes, NJ). Gels were solidified at 37°C. Ice cold SISgel, pH adjusted to 7.4 (0.8 ml), was layered onto each membrane of the 6-well cell culture inserts and allowed to solidify overnight at 37°C. Confluent cells growing on plastic were trypsinized with 1 ml 0.25% trypsin; 1.0 mM EDTA (Life Technologies). Trypsinized cells were resuspended in 2 ml of respective media and 500,000 cells aliquoted onto solidified SISgel or Matrigel discs. Two ml of medium (Minimal Essential Media containing 1× nonessential amino acids, L-glutamine and pyruvate, Life Technologies, Rockville, MD) containing 1% Fetal Calf Serum (Life Technologies) were layered beneath the transwell supports in 6-well plates such that an air bubble did not form. The cells were allowed to adhere to the gels for 72 hrs before the media were replenished. Cultures were grown for 14 days with media changes twice per week. The fraction of cells growing under the above conditions are approximately 15% when cells growing on plastic are allowed to become nearly confluent [13]. Cells were harvested from SISgel with 1 ml of 200 U/ml collagenase IV (Calbiochem, LaJolla, CA) following incubation at 37°C until the gel dissolved, approximately 1 hour. Cells were isolated from Matrigel with Matrisperse (BD Biosciences, San Jose, CA). The gels from 3 wells were removed and incubated with 30 ml of Matrisperse with shaking on ice for 30 min. The mixture was centrifuged, the supernatant was poured off and replaced by fresh Matrisperse until the process had been performed 3 times. After the final treatment, cells were centrifuged at 600 × g for 10 min and washed twice with cold PBS.

### Preparation of J82 cell lysates

J82 cell cultures grown on plastic, Matrigel or SISgel were harvested and placed in a final volume 2.5 mL of lysis-denaturing buffer. The composition of the lysis-denaturing buffer was: 6 M urea, 10% glycerol, 2 M thiourea, 50 mM Tris-HCl (pH 8.2 at 10°C), 5 mM Tris (2-carboxyethyl) phosphine hydrochloride, 2% (w/v) n-octylglucoside and 1 mM protease inhibitor cocktail (all components from Sigma-Alrich, St. Louis, MO). The samples were stored at -80°C until processing. To lyse the cells, the samples were thawed and then vortexed for 30 sec. Next each cell lysate was centrifuged at 20,000 × g for 60 min at 18°C. The each supernatant was removed and transferred into a PD10 column (Amersham Bioscience, Mountain View, CA), where the lysis buffer was exchanged with Protein Fractionation Start Buffer (Beckman Coulter, Inc., Fullerton, CA). Although the compositions of this buffer and the Elution Buffer (see below) are proprietary, similar formulations have been described [14]. The protein concentration was then determined by the bicinchoninic acid assay (MicroBCA, Pierce, Rockford, IL) with a DU800 spectrometer (Beckman Coulter, Inc.).

### Protein fractionation

The proteins from the three samples were fractionated by pI and hydrophobicity with an automatic two-dimensional liquid chromatography system, the ProteomeLab™ PF 2D Protein Fractionation System (Beckman Coulter, Inc.). This is a commercial version of an instrument described previously [11,14]. The first dimension separated the Proteins are first separated by chromatofocusing (first dimension) followed by a second-dimension separation by reversed-phase chromatography.

In the first dimension, the chromatofocusing column (HPCF-1D column, 250 × 2.1 mm, Beckman Coulter) was used at ambient temperature with a flow rate of 0.2 ml/min. Before injection, the column was equilibrated with the Protein Fractionation Start Buffer (pH 8.5, Beckman-Coulter, Inc. Fullerton, CA) for 130 min. The total mass of each sample type injected on the protein fractionation system was the following: 2.1 mg for cells grown on plastic; 4.4 mg for cells grown on SISgel; and 4.4 mg for cells grown on Matrigel. The first-dimensional separation was started with the injection of sample, which began the execution of the following steps automatically. The start buffer was pumped through the column for the first 20 min to elute proteins with pI value above 8.5. After 20 min, the pH gradient from 8.5 to 4.0 was started by introduction of the Eluent Buffer (pH 4.0). After the end of the pH gradient (115 min), a 1 M sodium chloride (Spectrum, Gardena, CA) solution was used to remove proteins with pI values below 4.0 from the column for 45 min. The pH of the effluent from the first dimension column is monitored continuously with an in-line pH meter. Within the reproducibility of the pH, it serves as a measurement of the fundamental molecular property of pI of eluted proteins. The final step was to rinse the column with water for 45 min. During the first-dimension separation, fractions were collected at every 0.3 pH units during the pH gradient and at every 5 min when the pH value was constant. Both pH measurements and absorbance at 280 nm data were collected throughout the separation at a data rate of 1 Hz. A total of 30 first-dimension fractions were collected over a 3 hour period. At the end of the first-dimension separation, the second dimension separation was started.

The second dimension used a high-performance, non-porous C-18 reversed-phase column with a flow rate of 0.75 ml/min at 50°C [15]. A gradient was formed using 0.1% trifluoroacetic acid (TFA; J.T. Baker Phillipsburg, NJ) in water and 0.08% TFA in acetonitrile (Burdick & Jackson, Muskegon, MI). The gradient was 0–100% of 0.08% TFA in acetonitrile over 30 min. The proteins were detected by absorbance at 214 nm with data collected at 5 Hz. The first-dimension fractions were analyzed by injecting 200 μl of each onto the second-dimension column. In mapping mode, second dimension fractions were not collected. The total time to run the first dimension fractions was about 48 h. Selected first-dimension fractions were re-run, and fractions were collected at intervals of 15 sec., 187.5 μl, between 4–24 minutes of the run for subsequent mass spectrometry analysis. A total of 80 fractions was collected for each second-dimension separation. Chromatograms were visualized either with ProteoVue, which displays a single chromatogram, or DeltaVue, which allows comparison of two chromatograms. Both are provided by Beckman Coulter.

### Mass spectrometric analysis

Peaks of interest were identified by examination of the proteomes of the 3 samples and selecting peaks that were unique to one sample. In order to afford the best chance for identifying proteins, the entire fraction was digested with sequencing grade modified trypsin (Promega, Madison, WI) at a 30:1 ratio of protein to trypsin using the integrated absorbance of the peak as a guide to protein content. The fractions were lyophilized to dryness prior to trypsinization. The proteins were dissolved in 100 μl of 50 mM NH_4_HCO_3 _followed by the appropriate volume of trypsin, and digestion was carried out overnight at 37°C. After digestion, liquid was removed by SpeedVac (Savant Instruments, Farmingdale, NY), and the dried samples were stored at -80°C until analysis. Each sample was dissolved in 10 μl of 0.2% trifluoroacetic acid and purified with a reversed-phase ZipTip (Millipore, Billerica, MA) as recommended by the manufacturer. The eluted tryptic digest of a fraction (0.5 μl) was mixed with 0.5 μl of matrix solution [2% (w/v) 2,5-dihydroxybenzoic acid in 50% (v/v) acetonitrile/0.1% (v/v) trifluoroacetic acid] and spotted on a stainless-steel MS sample plate. Peptide mass fingerprinting (PMF) analysis was performed by Voyager Elite MALDI-TOF MS (PerSeptive Biosystems, Framingham, USA). CID fragmentation of tryptic peptides was performed using MALDI-QIT-TOF MS (AXIMA QIT; Shimadzu/Kratos, Manchester, UK).

The peptide mass fingerprinting data were submitted to MASCOT peptide mass fingerprint program (Matrix Science, London, UK) [16] in order to obtain protein candidates for each fraction analyzed. Database searches were performed against the National Center for Biotechnology Information (NCBI) nonredundant database using the following parameters; (1) the protein database under *Homo sapiens *(2) unlimited protein molecular weight and pI ranges, (3) presence of protein modifications such as methionine oxidation, protein N-terminus acetylation, and pyroglutamic acid, and (4) peptide mass tolerance of ± 0.25 Da. After matching experimental peptide mass values against predicted peptide masses of each entry in the database, MASCOT calculates the probability based MOWSE score that is a measure of the statistical significance of the first protein candidate, and scores ≥ 67 represent p < 0.05 in the case of *Human *proteome. In order to confirm identities of protein candidates acquired in our peptide mass fragment analyses, the MS/MS data of several proteins were analyzed by MASCOT MS/MS ion search program (Matrix Science, London, UK). The database search parameters were the same as those used for peptide mass fragments except that a mass tolerance of ± 0.5 Da was set for precursor ions and ± 2.0 Da for fragment ions. We subjected 2–3 peptides per protein to MASCOT MS/MS ion search. We considered the confirmation to be positive when a significant MOWSE score (p < 0.05) was generated (1) individually from all the peptide fragments analyzed or (2) from the combined MS/MS data of the peptides and (3) matching fragment sequences spanned the entire sequence of the intact protein chain, that is the matching sequences were not all found near one terminus or solely in the middle.

### Gel electrophoresis of fractions

In order to compare the ease of PMF with LC/LC fractions as compared to analysis of gels, a set of peaks spanning the range of 0.2 to 0.025 AU was selected. The entire fraction or fractions containing a peak (140 or 280 μL, if the peak was distributed into two fractions) was lyophilized and dissolved in 10 μL of denaturing buffer. 1 μL of diothiothreitol and 2.5 μL of tracking dye were added and the entire volume was transferred to a 4–12% Tris-glycine gradient gel. Separation was at 90 V for 3 h. followed by Coomassie Blue or silver staining (BioRad, Hercules, CA).

### Pathway analysis

GenBank accession numbers for identified proteins were obtained via Entrez Gene [17]. Biologically relevant networks were assembled from genes identified on Matrigel, SISgel and plastic by using Ingenuity Pathways Analysis (IPA). This web-based application (Ingenuity Systems, [18]) enables the visualization and analysis of direct and indirect interactions among genes. Each gene identifier was mapped to its corresponding gene object in the Ingenuity Pathways Knowledge Base. Genes were not weighted by expression levels, and biological networks were built on this assumption.

## Results and discussion

**Figure 1:**
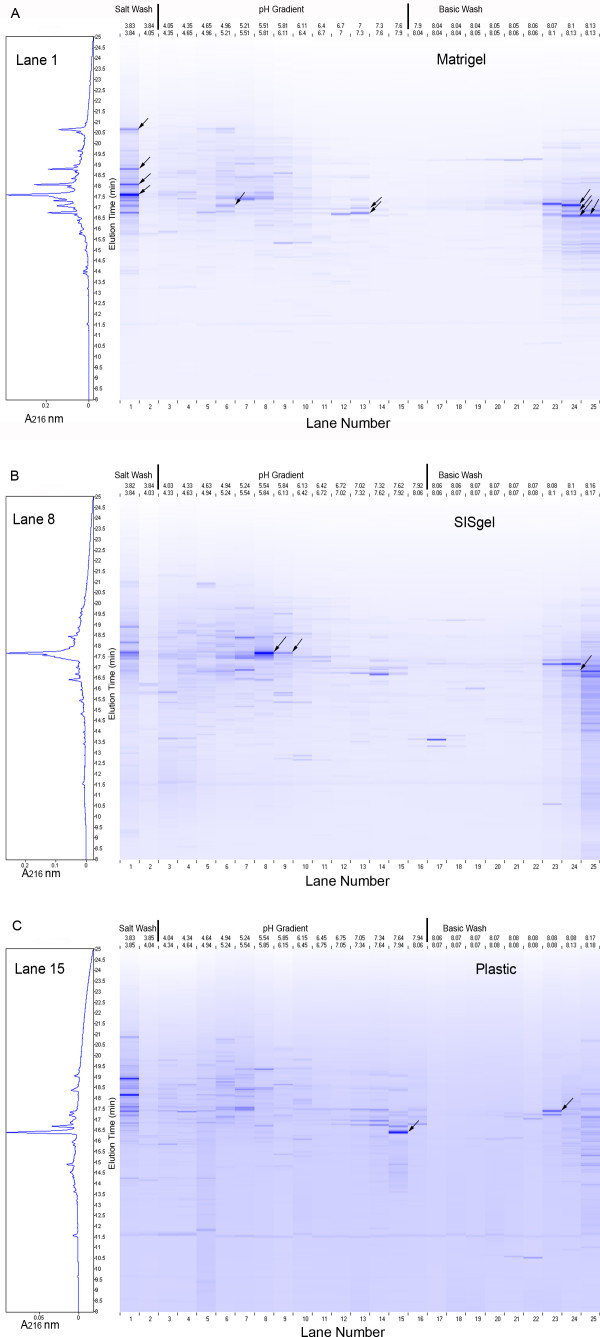
***"Gel-view" proteomic display of J82 cells cultured on Matrigel (A), SISgel (B) and plastic (C)***. Due to the current software configuration, the elution is from right to left, with lanes 17–25 showing proteins eluted during the base wash, lanes 2–16 showing proteins eluting during the pH 8.3-4.0 gradient, and lane 1 showing proteins eluting in the 1.0 M NaCl wash. The Y axis shows the retention times of the second dimension between 10 and 24 minutes. The software presents the elution pattern as a simulated gel-view in which the color intensity is proportional to the absorbance. On the left of each display is shown a chromatographic view of one lane from which fractions were obtained for MS analysis presented as A_216 _vs retention time. The black arrows identify fractions taken for MS analysis and reported in Table 1.

The proteomes of the J82 bladder cancer cells grown, on Matrigel, SISgel and on plastic in conventional tissue culture are displayed in Fig. 1. Although the actual data are in the form of chromatographic traces of absorbance at 214 nm, for convenience in displaying and comparing chromatograms, a flat "gel-view" is used instead. Each "lane" corresponds to a first dimension fraction collected by pH and separated by reversed phase chromatography. Therefore the experimental pI of all proteins eluting within that fraction is specified experimentally by the pH range over which the fraction was collected. Retention time increases from bottom to top in this display. The absorbance of each peak is indicated by the intensity of the artificial band constructed by the software. To provide a comparison of the "gel view" and a conventional chromatographic display, one "lane" is also presented as an absorbance profile on the left. The "Lanes" are numbered from the most acidic to the most basic, which is the inverse of the actual elution order. The number of distinct peaks detected was 1345 from the cells grown on plastic, 1582 from the cells grown on Matrigel and 1984 from the cells grown on SISgel.

The display of the proteome falls into 3 regions. First is the basic wash, where the separation on the first dimension is by unknown parameters. Several peaks at the same retention time are seen in adjacent lanes, indicating the resolution of the first dimension separation above pH 8.3 is probably minimal. Next is the region of the pH gradient, where the separations by pI are generally clear, as demonstrated by the presence of numerous bands. Some bands are observed with identical elution times in adjacent lanes, which may indicate the separation of fractions for the first dimension splits an individual protein between two fractions. Finally is the salt wash, which also presents a very complex set of fractions indicative of minimal separation in the first dimension.

The first step in further analysis was to identify some of the proteins represented in one of the samples but not in either of the other two. These unique components should be biologically the most interesting and would be expected to be enriched in post-translational modifications. The peaks selected for further analysis are indicated by the arrows. The proteins that could be identified to a confidence of P < 0.05 are listed in Table 1. The proteins can be matched with the proteomic displays by the pH of the first dimension fraction (top of displays in Fig. 1) and retention time of the second dimension according to the arrows. All of the peaks selected yielded an identifiable protein with the exception of the abundant protein in lane 15 (plastic). Although the fraction provided a clear mass fragmentation, it did not match any known protein.

**Table 1:** Identification of several proteins unique to one or more samples. Only those for which a MOWSE score of >65 (p = 0.05)for PMF or >34 (p = 0.05) for MS/MS and for which matching sequences spanned the entire sequence are reported. The experimental pI is the measured pH value for the first dimension fraction. The # Seq. Matched represents the number of fragments matching peptide sequences.

Exp. pI	RT 2^nd ^Dim. (min)	Gene Symbol	NCBI Accession Number	Sequence MW/pI	MOWSE Score (PMF)	Coverage % (# Seq. Matched)	MOWSE Score (MS/MS)
**Cells grown on Matrigel**							
<4.0	17.560	ER60 HSPA5 VIME	2245365 16507237 37852	57245/5.88 72431/5.07 53724/5.06	81 68 86	29 (13) 23 (11) 28 (13)	94
<4.0	18.027	HSPA5	16507237	72431/5.07	120	29 (17)	
<4.0	18.777	HSPA8 HSPA5 ENOA	24234686 16507237 1167843	53626/5.62 72431/5.07 47566/7.01	103 136 96	25(7) 22(11) 17(8)	41 36
<4.0	20.687	HSP60	49522865	61229/5.71	99	20(8)	93
4.63–4.94	17.167	ATM	1497931	356785/6.37	74	9 (19)	
7.02–7.32	16.740	H2B.1	1568551	13928/10.3	106	57(9)	48
7.02–7.32	16.998	RPL9 RPS18	13278765 75517910	21992/9.96 17708/11.0	101 73	53 (8) 38 (9)	78 44
>8.3	16.597	APC	68533057	314407/8.03	70	9 (18)	
>8.3	16.687	ALDOA	4557305	39964/8.33	70	26 (7)	46
>8.3	17.094	GAPDH	31645	36244/8.26	191	66 (16)	64
>8.3	16.634	RUVBL/CHTF18	14336725	130269/9.51	72	16 (13)	
**Cells grown on SISgel**							
>8.3	16.774	RSNL2	48257203	60839/9.07	70	23 (11)	
5.54–5.84	17.667	AKR1B1 ATPB	13529257 28931	36298/6.82 34026/4.90	155 96	49 (14) 25 (6)	74 59
5.54–5.84	17.695	AKR1B1 ATPB	13529257 28931	36298/6.82 34026/4.90	166 90	48 (17) 28 (7)	68 79
5.94–6.13	17.667	AKR1B1 ATPB	13529257 28931	36298/6.82 34026/4.90	98 48	55 (13) 22 (3)	51
**Cells grown on plastic**							
>8.3	17.407	GAPDH	31645	36244/8.26	192	56 (16)	68

**Figure 2:**
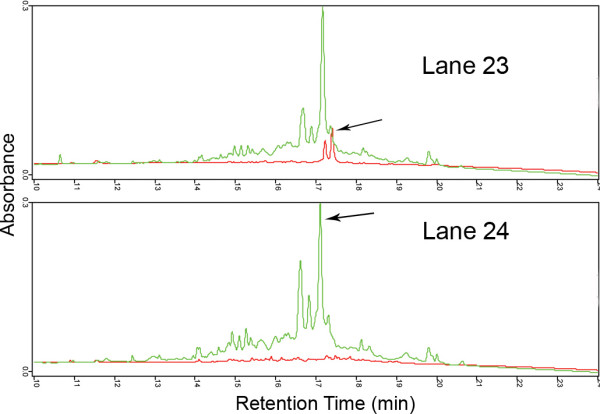
***DeltaVue comparison of 2^nd ^dimension fractionations of basic first dimension fractions fractions***. Lane 24 and Lane 23 showing different properties of glyceraldehyde 3-phosphate dehydrogenase in cells grown on plastic and on Matrigel. The arrows indicate the identified proteins. Green = cells grown on Matrigel, Red = cells grown on plastic.

Clear differences in the proteomes are evident. In examining the fractions in which proteins have been identified, the cells growing in Matrigel express a number of chaperone molecules not seen in the other two samples. Additionally, because many of the proteins show experimental pI's different from the sequence pI, post-translational modification seems to represent a major theme in these uniquely expressed proteins. Because post-translational modification represents a major means of regulating proteins, these are likely to be key molecules related to the difference in phenotype. Of interest is the identification of glyceraldehydes 3-phosphate dehydrogenase (GAPDH) at a different retention in the second dimension separation. This is not an artifact, as is shown in Fig. 2, which compares the second dimension separations of the basic pH fractions, Lanes 23 and 24, that eluted before the pH gradient, from the cells grown on plastic and Matrigel. Lanes 23 and 24 from Matrigel both contain a large peak eluting at 17.094 min that was identified as GAPDH (green trace), On plastic the cells also expressed a different form that eluted at 17.407 min and corresponded to a small peak in the proteome expressed in Matrigel. The difference in retention time, 0.313 min (18.7 sec), is significant. In cells grown on SISgel, the peak was not seen in Lane 23. However, two peaks corresponding to the adjacent peaks seen in the Lane 24 from cells grown on plastic were seen, except that the height of the peak with the 17.094 min retention time was higher than the one eluting at 17.407 min that was identified by MS. These results also indicate that the resolution of proteins in the first dimension at >pH 8.3 is less than in the pH gradient.

**Figure 3:**
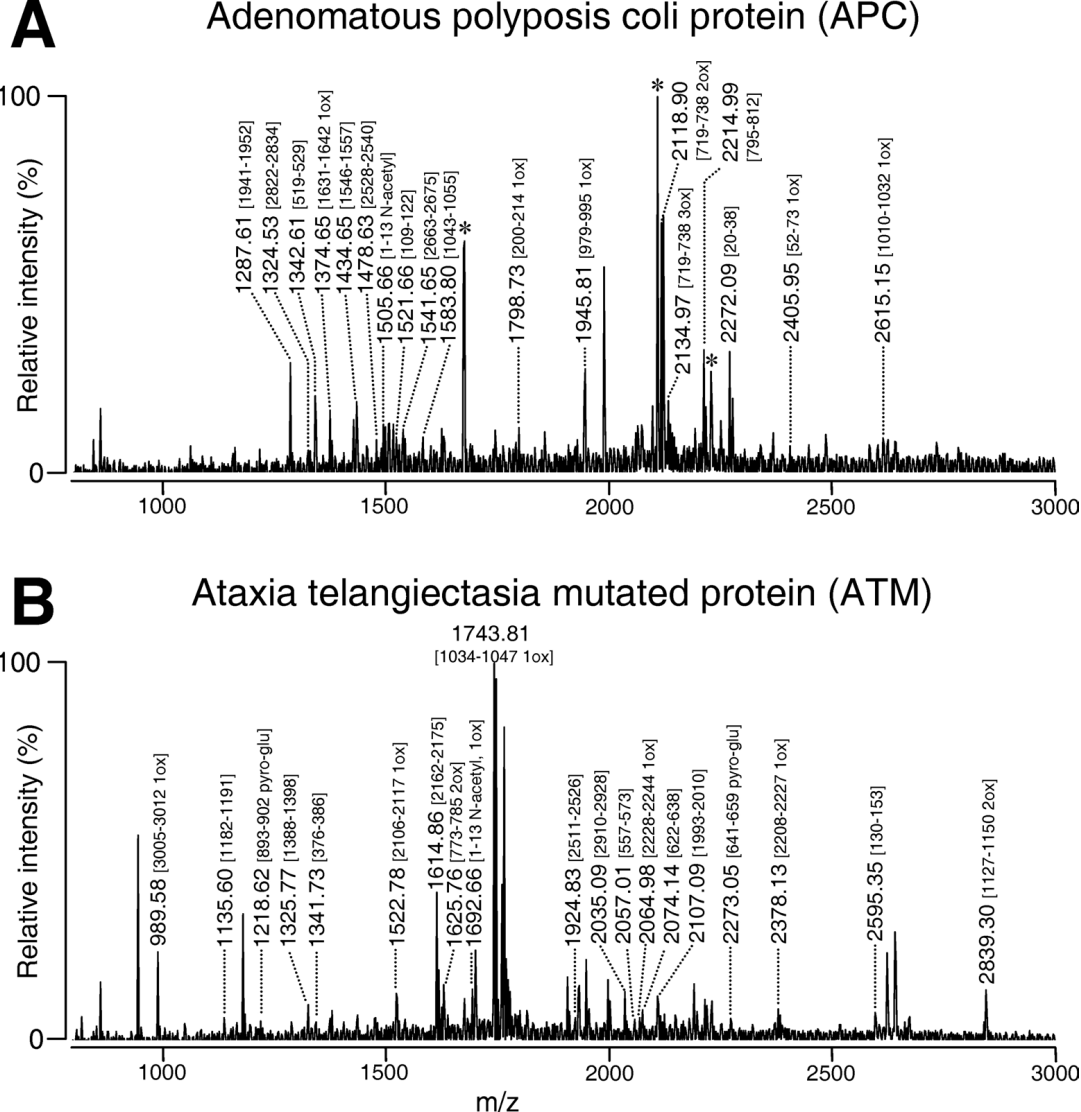
***Peptide mass fingerprinting of APC and ATM proteins***. Fragments matching sequences in the protein are indicated along with the amino acid numbers. Note that sequences from the entire protein are matched, indicating that the intact protein is identified. The peaks marked with an asterisk in A. match Aldolase A, which elutes 0.09 min later than APC. The unassigned peaks in ATM do not match a single protein.

Of particular interest was the finding of two, very large proteins with sequence molecular weights exceeding 300 KDa. The peptide mass fingerprints of these proteins are shown in Fig. 3. The proteins were identified as APC (2843 aa) and ATM (3066 aa). As is seen from the fragments identified, the entire protein sequence was spanned, which argues against the proteins being proteolytically cleaved fragments of lower molecular weight.

**Figure 4:**
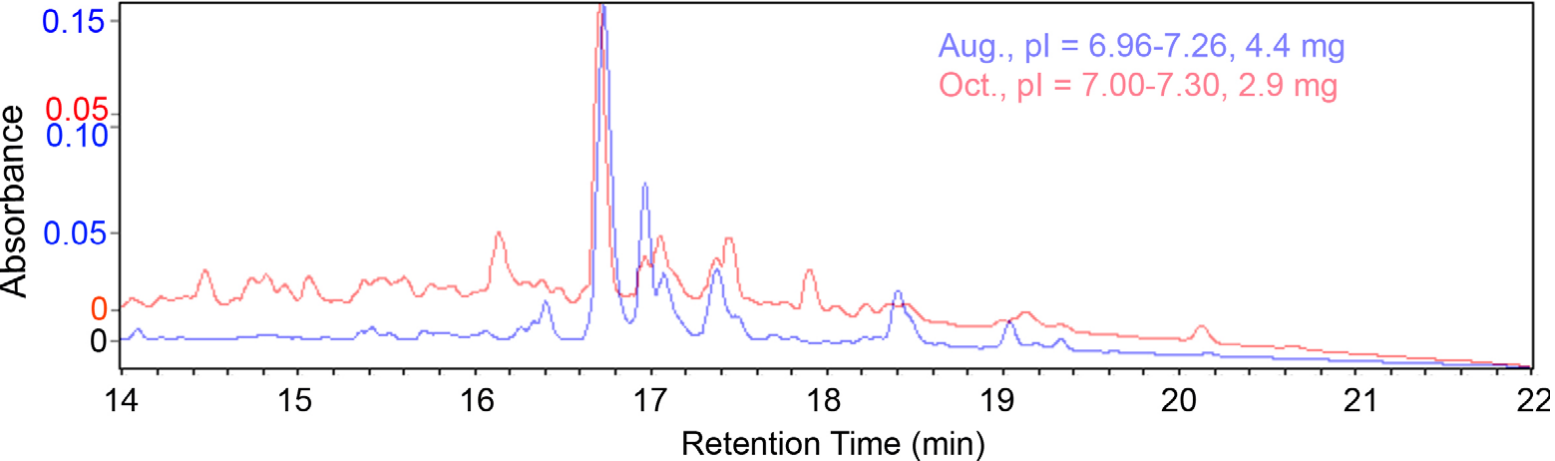
***Reproducibility of separations on the PF2D system***. Equivalent first dimension fractions of separate cultures grown on Matrigel analyzed approximately 3 months apart. The peak heights were normalized to the same relative size to show the similarities in shape. The actual absorbances are shown on the left, color-coded.

The reproducibility of separations was also evaluated by comparing the fractionation of two separate protein preparations made at different times and run about 3 months apart, as shown in Fig. 4. Because the pH gradient had been modified slightly, no two fractions showed identical pI ranges. However, one pair showed only an 0.02 pH unit difference. The retention times of the main peaks were reproducible to within ± 5 seconds in the second dimension. The ratios of some peaks were reversed, presumably reflecting biological variability in those proteins, although all the peaks will require identification in order to demonstrate this more clearly. The presence and absence of some peaks could be due to the small difference pH ranges included. The number of peaks counted in different fractions analyzed 3 months apart was virtually identical, in spite of a difference in the amount of material injected. The total number of peaks observed in cells grown on Matrigel was 1582 (4.4 mg protein) vs 1525 (2.9 mg protein) and 1999 (4.4 mg protein) vs 1984 (2.9 mg protein) for cells grown on SISgel in samples run 3 months apart.

**Figure 5:**
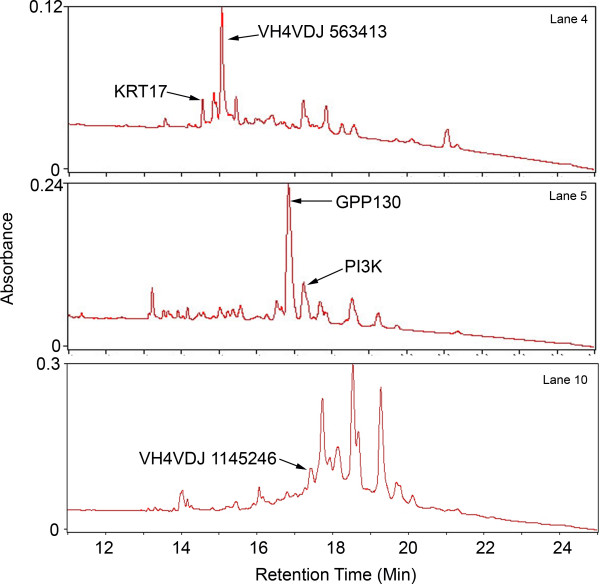
Selection of peaks of different sizes from J82 cells growing on plastic identified in Table 2.

One potential advantage of the chromatographic approach is that the fractions are presented in liquid with a simple solvent, as opposed to a within a gel. We attempted a preliminary comparison of sensitivity of gel-based and chromatographic fractionation methods. We selected a series of peaks of different peak heights ranging from about 0.2 AU in size down to 0.025 AU. In general, only peaks with heights of 0.1 AU or more showed any discernible bands on polyacrylamide electrophoresis when stained with Coomassie Blue. Very faint bands with silver stain were discernible down to about 0.05 AU analyzing the entire sample. The results of peptide mass fingerprinting of five such randomly selected fractions selected from cells grown on plastic are summarized in Table 2. As shown in Table 2, proteins forming small peaks in the range of 0.025 AU are easily identifiable using about 10% of the fraction.

**Table 2:** Identification of proteins as a function of peak height using in solution digestion. The lower MOWSE scores reflect the low molecular weights of two of the proteins and their most probable identification as antibody fragments.

Peak Ht (AU)	Gene Symbol and NCBI Accession No.	Seq. M/pI	MOWSE Score	Coverage% (Seq. Matched)
0.206	GPP130 2145095	81902/4.73	67	19(10)
0.114	VH4VDJ 563413	9534/9.17	51	71(4)
0.083	VH4VDJ 1145246	14616/5.58	56	61(5)
0.049	PI3K 2143260	192677/8.24	62	10(12)
0.025	KRT17 21754583	40577/4.90	70	30(10)

As with all proteomics techniques, however, more than a single protein was sometimes found in the fractions as collected, particularly those collected in the basic and salt wash regions. In part this reflected that the time-collected fraction width was greater than the width of homogeneous protein peaks and that some peaks do represent unresolved proteins. The option of collecting by automated peak detection would be helpful. The resolution of proteins is optimal in the pH gradient. The fractions eluting before and after the gradient are more complex, and the principles of separation on the chromatofocusing column in these regions are not completely understood [11,12,14,19]. However, given that 90% of the sample remains after MS, the possibility of a third dimension such as separation by molecular weight on gels, by LC [12,19,20], or even by MS [21] is certainly feasible if further resolution is needed. In addition, the possibility of MALDI-TOF on the intact protein is feasible, thereby allowing identification of the intact molecular weight.

**Figure 6:**
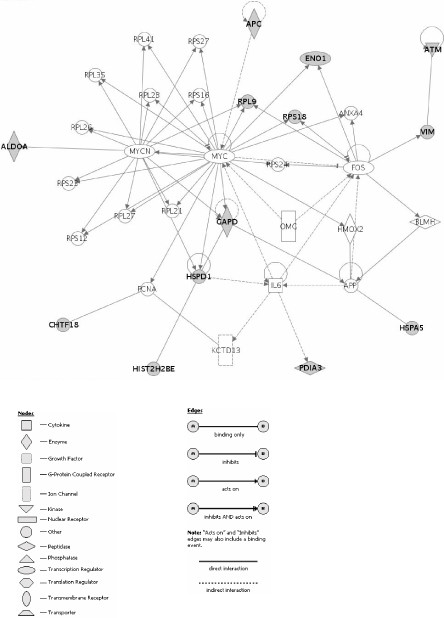
***Genes and relevant networks for Matrigel***. Focus gene/protein identifiers displayed as bold text. Proteins that were identified in samples are shown in gray color.

Pathway analysis of the proteins identified in Table 1 yielded several interesting findings. Of the 13 proteins that were uniquely found in Matrigel, all 13, including GAPDH, fit into a pathway involving MYC, as shown in Fig. 6. Although GAPDH is thought to be a simple glycolytic enzyme and "housekeeping gene" [22], it is involved in telomere shortening and may have other signaling roles as well [23]. This protein apparently bears different post translational modifications in cells grown on plastic and Matrigel. The MYC pathway also was recently identified as playing a key role in suppression of the malignant phenotype [24]. Based on the pI being much less than the pI calculated from the sequence, the histone H2B.1 protein likely was post-translationally modified in cells growing on Matrigel. Interestingly, although the other histones of the chromatin complex were reported to be phosphorylated and acetylated in K562 erythroleukemia cells, H2B was not [25]. On SISgel, only two unique proteins were identified (Table 1). That many of the unique proteins identified in cells grown on Matrigel fit into one network reinforces the suggestion made above that they are key players in regulating the phenotype, and that post-translational modification plays a major role in regulating the phenotype. Further identification of proteins that are differentially expressed among the three growth conditions will be required to fill in these pathways.

These preliminary findings support our hypothesis that the malignant phenotype is suppressed on SISgel. The two ribosomal proteins and the glycolytic enzymes aldolase and enolase found in cells grown on Matrigel most likely reflect the higher level of protein translation and glycolysis by malignant cells, whereas several others are associated with malignant functions. Interestingly, although aldolase reductase appears to be a metabolic enzyme, it also functions as a signaling molecule. In endothelial cells it has been reported to regulate TNF signaling and to upregulate cell adhesion molecules on the cell surface [26], a function that is consistent with a less malignant phenotype. Further work will require identifying a larger number of proteins in the fractions and assembling a picture of the pathways involved in the biology.

## Conclusion

Part of the power of the proteomics approach in general is that it is sensitive to post-translational modifications that represent the most important means of biologically modulating the activities of proteins involved in signaling pathways. Microarray techniques yield no information concerning protein modification and are silent concerning changes in protein concentration that are not associated with altered transcription. The differences in the actual pI and the sequence pI shown in Table 1 strongly suggests that some of these unique peaks may represent post-translational modifications that shift the chromatographic properties of particular proteins. The selection of proteins on the basis of being expressed uniquely in one sample is likely to be highly represented in post-translationally modified proteins. Chaperone-type proteins were up-regulated in the cells growing on Matrigel. Glyceraldehyde 3-phosphate dehydrogenase is apparently post-translationally modified in a different way, depending upon the matrix on which the cells are grown. Pathway analysis of the unique proteins showed that all those identified in cells grown on Matrigel fit into a pathway involving MYC. Those uniquely expressed on SISgel and plastic also suggested signaling pathways. Future work to identify all the differentially expressed proteins should shed further light on these pathways. As a tool for biological investigation, this system provides several advantages. The system is reproducible with respect to retention time and one parameter (pI) is a fundamental molecular property that is measured. In addition, as shown by finding large proteins such as APC (311 KDa) and ATM (357 KDa) the upper limit imposed by the necessity of entering a gel does not appear to be operant with chromatographic display. Third, the sensitivity with respect to MS identification using in solution digestion is excellent, and even small peaks (0.03 AU) are readily identifiable without loss in retrieving from a gel. The main limit to reproducibility appears to lie in the chromatofocusing, not the reversed phase separation. Resolution was highest in the fractions eluted during the pH gradient as opposed to those eluting during the pH 8.3 and salt washes.

## Abbreviations

AU: absorbance units

ECM: extracellular matrix

LC: liquid chromatography

MALDI-TOF: Matrix Assisted Laser Desorption/Ionization-Time Of Flight

MS: mass spectrometry

SIS: small intestine submucosa

## Competing interests

Dr. Hurst has a grant from Beckman Coulter, Inc. to support work using the PF2D instrument. Other than this, the authors declare that they have no competing interests.

## Contributions of authors

REH conceived the study, performed most of the data analysis and wrote the paper. KDK grew the cells and verified their phenotypes. MD performed Ingenuity Pathway Analysis and helped to finalize figures and the manuscript. NT and AS performed the mass spectrometric analyses and provided interpretations under the direction of HM, who also helped with the writing of the manuscript. RS helped with the pathway analysis and interpretation of networks. EB performed the PF2D analyses under the direction of MS, who assisted with the interpretation of the protein separations and assisted with the writing of the paper.
